# A Systematic Review of Scope and Quality of Health Economic Evaluation Studies in Vietnam

**DOI:** 10.1371/journal.pone.0103825

**Published:** 2014-08-14

**Authors:** Bach Xuan Tran, Vuong Minh Nong, Rachel Marie Maher, Phuong Khanh Nguyen, Hoat Ngoc Luu

**Affiliations:** 1 Johns Hopkins Bloomberg School of Public Health, Baltimore, Maryland, United States of America; 2 Institute for Preventive Medicine and Public Health, Hanoi Medical University, Hanoi, Vietnam; 3 Health Strategy and Policy Institute, Ministry of Health, Hanoi, Vietnam; Federico Ii University of Naples, Italy

## Abstract

**Introduction:**

The application of health economic evaluation (HEE) evidence can play an important role in strategic planning and policy making. This study aimed to assess the scope and quality of existing research, with the goal of elucidating implications for improving the use of HEE evidence in Vietnam.

**Methods:**

A comprehensive search strategy was developed to search medical online databases (Medline, Google Scholar, and Vietnam Medical Databases) to select all types of HEE studies except cost-only analyses. Two researchers assessed the quality of selected studies using the Quality of Health Economic Studies (QHES) instrument.

**Results:**

We selected 26 studies, including 6 published in Vietnam. The majority of these studies focused on infectious diseases (14 studies), with HIV being the most common topic (5 studies). Most papers were cost-effectiveness studies that measured health outcomes using DALY units. Using QHES, we found that the overall quality of HEE studies published internationally was much higher (mean score 88.7+13.3) than that of those published in Vietnam (mean score 67.3+22.9). Lack of costing perspectives, reliable data sources and sensitivity analysis were the main shortcomings of the reviewed studies.

**Conclusion:**

This review indicates that HEE studies published in Vietnam are limited in scope and number, as well as by several important technical errors or omissions. It is necessary to formalize the process of health economic research in Vietnam and to institutionalize the links between researchers and policy-makers. Additionally, the quality of HEE should be enhanced through education about research techniques, and the implementation of standard HEE guidelines.

## Introduction

Health economic research has been increasingly utilized over the past few decades, particularly in developed countries, in order to improve the efficiency of healthcare spending. Health interventions, particularly medical technology and pharmaceuticals, are enormously expensive, and thus many countries have implemented formal health economic evaluation processes, such as Health Technology Assessments (HTA), as a standard tool for selecting and implementing interventions [6], [37]. However, despite the greater gap between resources and need in developing countries, these countries lag far behind in the production of health economic research and the translation of research findings into health care policies [1].

Vietnam is a country that has yet to formalize a health economic evaluation system, although its healthcare system faces rising costs and dwindling resources. Since the market-oriented reforms of the 1980s, hospitals in Vietnam have turned to privatization, including the introduction of user fees and private hospitals, clinics, and pharmacies [1]. As has been observed in several other countries, including South Korea, Taiwan, and China, this fee-for-service system often leads to inefficiency and waste, as it incentivizes medical practitioners to overprescribe drugs and technology. Additionally, Vietnam’s healthcare spending is likely to increase, as its disease burden has shifted away from infectious diseases to more chronic, non-communicable diseases, which are often much more expensive to treat. Despite the introduction of social insurance to help offset the cost of healthcare, today 70–80% of financing for health services come from out-of-pocket (OOP) payments [1]. Moreover, while healthcare spending has been supplemented by foreign aid, this funding is starting to decrease, as Vietnam has just reached the lower middle-income level. It is, therefore, very necessary for the health sector to implement effective measures toward a sustainable and highly efficient health care system.

Several countries in Southeast Asia, including Thailand, Malaysia, and Taiwan, have established a Health Technology Assessment process in order to use healthcare resources more efficiently [35], [36]. HTA provides evidence on consequences of polices on health technologies by reviewing and assessing their safety, efficacy, patient-reported outcomes, cost-effectiveness, and social, legal, ethical and political impacts. However, despite the potential of HTA in reducing healthcare spending, the use of health economic research in policy is often constrained by several factors, including lack of communication between researchers and policy-makers, research that is irrelevant to policy-makers, and poor study quality. With this in mind, this paper seeks to explore the scope and quality of health economic evaluation (HEE) studies that currently exist about Vietnam, in order to better understand what research is available for such resource allocation decisions and how it can be strengthened. We did this by conducting a systematic review of all HEE studies published about Vietnam, both within the country and internationally. By analyzing the strengths and shortcomings of existing health economic evidence, we recommend potential areas of improvement in the field of health economic research in Vietnam.

## Materials and Methods

### Search strategy and data sources

A comprehensive search strategy was developed to search for published health economic evaluation studies about Vietnam. The systematic search was performed in March 2013 and included all results up to 2013. For English published articles, we searched Medline and Google Scholar with combination of free-text keywords, including “cost”, “economic”, “expenditure,” and “Vietnam”. For Vietnamese published studies, we searched through a variety of Vietnamese medical databases, including the Vietnam Central Medical Library (VCML), the Hanoi Medical University Library, the Hanoi School of Public Health Library, the Hanoi University of Pharmacy Library, and the Health Strategy and Policy Institute Databases. The VCML was particularly useful, as it is the biggest database center of the Vietnamese official national medical database, managed by the Ministry of Health. (The search terms used are shown in **Table 1**).

**Table 1:** Search strategy.

Data sources	Keywords
**PubMed**	(((((((((cost) OR costing) OR financial)OR economic) OR policy) OR cost-effectiveness)OR cost utility) OR cost benefit) OR cost minimization)AND Vietnam
**Google Scholar** **and Google.com**	“cost effectiveness” or “cost utility” or“cost benefit” or “cost minimization”and “Vietnam”
**Vietnamese** **data**	“chi phí” ho 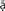 c “tài chính” ho 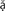 c “kinh t 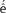 ”ho 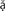 c “chi phí-hiêu qu 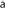 ” ho 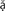 c “chi phí-l 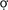 i ích”ho 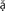 c “chi phí – th 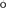 a d 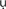 ng”
**Vietnamese** **database**	The Vietnam Central Medical LibraryThe Hanoi Medical University LibraryThe Hanoi School of Public Health LibraryThe Hanoi University of Pharmacy LibraryThe Health Strategy and Policy Institute Databases

### Study selection and inclusion/exclusion criteria

Two researchers independently selected the studies for review via a two-stage screening process. First, the titles and abstracts of all the initial search results were reviewed. Based on the titles and abstracts, potentially relevant articles were selected for further review, which involved examining the content of their full text. All types of health economic evaluations that analyzed costs and consequences of one or more interventions were eligible for inclusion in the study, including cost-effectiveness, cost-utility, cost-benefit, and cost-minimization analyses. Cost-only analyses were excluded. In addition, we screened reference lists and contacted authors of selected studies for more potential papers. To avoid publication bias, we contacted all Vietnamese and international researchers who were active in health economics research in Vietnam to inform them the review and ask them if there is any study their institution has done or they were involved that falls into the scope of the review. In addition, we search purposively HEE studies in Vietnam using search engine such as Google.com. Besides, we used a standardized tool for data extraction and study quality assessment to avoid missing in reporting the content of the selected studies. The complete inclusion/exclusion criteria are shown in **Table 2**.

**Table 2:** Inclusion/Exclusion criteria.

Inclusion	Exclusion
• Full health economic evaluations:comparing both costs and outcomesof two or more interventions.	• Cost analysis studies, notcomparing costs and outcomesof interventions
• Partial economic evaluations:analyzed costs and outcomesof one intervention	• Not economic evaluation studies,incl. impact, financial, or healthexpenditure studies
	• Data not from Vietnam
	• Not published in English or Vietnamese

### Data extraction

We developed a standardized extraction form to collect data from all eligible studies. Data extraction was performed by the first researcher (N.M.V) and verified by the second researcher (B.X.T). The data extraction form was divided into three sections: general information, study details, and results & findings.

### Quality appraisal

The quality of selected studies was assessed and graded using the Quality of Health Economic Studies (QHES) instrument. The QHES has been proven to be a simple, consistent and suitable scale to measure the quality of health economic evaluation studies, especially cost-effectiveness studies [3]. It consists of 16 criteria in the form of “yes or no” questions that were selected by 8 experts in health economics [4]. Each question has a weighted point value ranging from 1–9, which are used to generate a summary score from 0–100 [4]. This QHES tool was used by two independent researchers to rate the quality of the selected studies. Since no standardized interpretation of the QHES exists, we set a score of 75 to 90 as good quality and a score of 90 and above as excellent quality.

### Data analysis

Descriptive statistical analysis, including frequency and percentages, was used to describe the characteristics of the studies. Health outcomes were measured by incremental cost-effectiveness ratio (ICER): cost per life year saved, cost per case averted, cost per DALY (disability-adjusted life year), and cost per QALY (quality-adjusted life year). These costs were adjusted for inflation as measured by the 2012 World Bank Consumer Price Index (CPI). Studies which reported cost in Vietnamese Dong were converted to US Dollar at the year of research.

The WHO Guide to Cost-Effectiveness Analysis (WHO-CHOICE) categorizes interventions as “highly cost effective” when the ICER (measured as cost per DALY averted) is less than GDP per capita, “cost-effective” when the ICER is between one and three times GDP per capita, and “not cost-effective” when the ICER is more than three times higher than GDP per capita [5]. Following this guideline, we assessed the cost-effectiveness of interventions included in the selected studies by comparing their reported ICERs with GDP per capita in Vietnam, which in 2012 was approximately 1,500 USD.

## Results

### Search results

**Figure 1:**
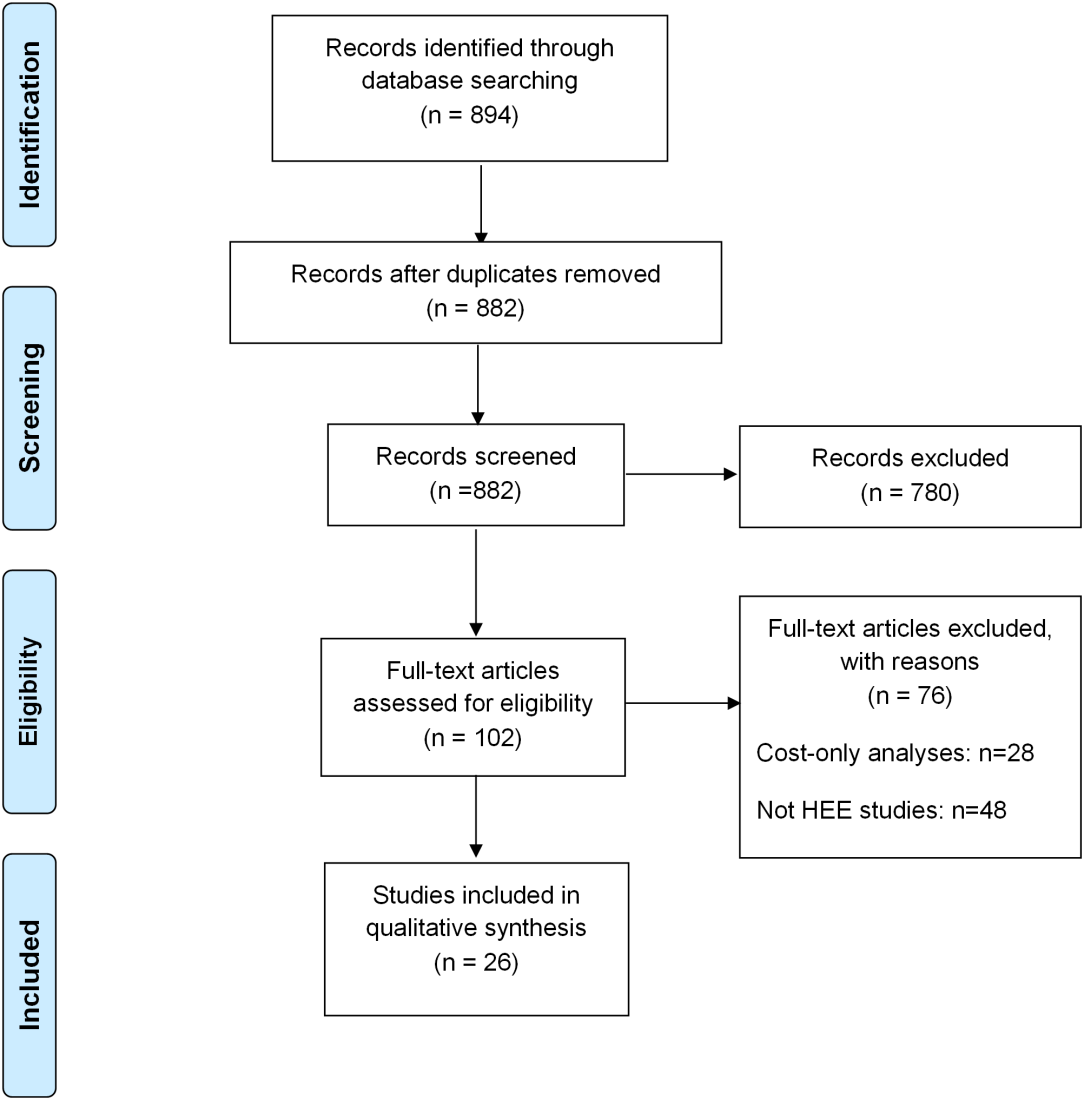
PRISMA flow chart of study selection.

We conducted a search of potential articles through MedLine, Google Scholar, and a variety of Vietnamese databases. After identifying a large number of potential articles using key search terms, we used a two-stage screening process to select 26 papers for inclusion (see PRISMA flowchart in **Figure 1**). A total of 882 articles were identified in the initial search, including 758 results from MedLine, 52 results from Google Scholar and 72 results from several Vietnamese databases. By applying the inclusion/exclusion criteria for titles and abstracts in the first stage screening, 780 articles were excluded. We reviewed 102 full-text papers at the second stage and rejected an additional 76 studies. After this screening process, we were left with 26 studies to analyze in this review [10]–[34], [38], including 20 published internationally [10]–[28], [38] and 6 published in Vietnam [29]–[34]. A total of 12 papers were written by Vietnamese corresponding authors. Most articles excluded from this review were either not health economic evaluations or did not use data from Vietnam. (See **Table S1** for profile of selected studies).

### Scope of health economic evaluation studies in Vietnam

The studies were analyzed for the type of diseases they evaluated (**Table 3**). More papers presented research about infectious diseases (14 studies) than non-infectious diseases (12 studies). Among studies about infectious diseases, the most common topic was HIV (5 studies), while a high percentage of the non-infectious disease papers focused on cancer (4 studies).

**Table 3:** **Classification of studies by disease type studied.**

Type of diseases		N	%
**Infectious** **diseases**	HIV	5	19.23
	Hepatitis	2	7.69
	Diarrhea	2	7.69
	Malaria	1	3.85
	Typhoid	1	3.85
	Encephalitis	1	3.85
	Liver fluke	1	3.85
	Others	1	3.85
**Non-Infectious** **diseases**	Cancer	4	15.38
	Smoking	2	7.69
	Exsanguinate	2	7.69
	Reproduction	1	3.85
	Cardiovascular disease	1	3.85
	Mental Health	1	3.85
	Cerebral hemorrhage	1	3.85

The scope, methods and measures of selected studies are shown in **Table 4**. We found that the majority of health economic evaluation studies in Vietnam were cost-effectiveness analysis studies, 12 of which reported ICER by DALY/QALY units. In contrast, we identified only one cost-benefit study. The type of interventions evaluated included both treatment (n = 9) and prevention (n = 14) interventions, although only three studies evaluated both types in one paper. Most of the studies calculated costs from the perspective of the health care system (n = 11), while 7 studies examined costs from the perspective of society; only 1 study estimated cost from the perspective of service users, and 1 examined the perspective of third-party payers. Nine studies did not state the perspective of cost-effectiveness analysis at all.

**Table 4:** The scope, methods and measures of selected studies.

		ALL (n = 26)		Vietnamese corresponding authors						International corresponding authors (int journals) (n = 14)	
				International journals (n = 6)		Vietnamese journals (n = 6)		ALL (n = 12)			
		N	%	N	%	N	%	N	%	N	%
**Type of health** **economic evaluation**	Cost-effectivenessanalysis	25	96.15	5	83.33	6	100	11	91.67	14	100
	ICER: cost perDALY/QALY	12	46.15	4	66.67	2	33.33	6	50.00	6	42.86
	Cost savings	2	7.69			1	16.67	1	8.33	1	7.14
	Cost-benefit analysis	1	3.85	1	16.67			1	8.33		
**Subjects**	Health programs	16	61.54	4	66.67	3	50	7	58.33	9	64.29
	Health systems	10	38.46	2	33.33	3	50	5	41.67	5	35.71
**Sectors**	Preventive	14	53.85		16.67	6	100	7	58.33	7	50
	Treatment	9	34.62	2	33.33			2	16.67	7	50
	Both preventiveand treatment	3	11.54	3	50			3	25.00		
**Perspective**	Society	7	26.92	1	16.67			1	8.33	6	42.86
	Health system	11	42.31	3	50			3	25.00	8	57.14
	Third-party payer	1	3.85							1	7.14
	Service users	1	3.85	1	16.67			1	8.33		
	Not stated	9	34.62	1	16.67	5	83.33	6	50.00	3	21.43
**Cost included**	Direct costs	24	92.31	5	83.33	6	100	11	91.67	13	92.86
	Investment costs	12	46.15	3	50	1	16.67	4	33.33	8	57.14
	Recurrent costs	24	92.31	5	83.33	6	100	11	91.67	13	92.86
	Indirect costs	8	30.77	1	16.67	3	50	4	33.33	4	28.57
	Not stated	2	7.69	1	16.67			1	8.33	1	7.14
**Cost analysis** **approach**	Activity-based costing	7	26.92			5	83.33	5	41.67	2	14.29
	Top-down costing	3	11.54	1	16.67	1	16.67	2	16.67	1	7.14
	Bottom-up costing	9	34.62	3	50			3	25.00	6	42.86
	Not stated	10	38.46	2	33.33			2	16.67	8	57.14
**Health** **outcomes**	Cost perLYS/case averted	12	46.15	1	16.67	4	66.67	5	41.67	7	50
	Cost perQALY/DALY	12	46.15	4	66.67	2	33.33	6	50.00	6	42.86
	Money units	3	11.54	1	16.67	1	16.67	2	16.67	1	7.14
**Sensitivity** **Analysis**	One-way	13	50.00	1	16.67	3	50	4	33.33	9	64.29
	Multi-way	2	7.69							2	14.29
	Probabilistic	4	15.38	3	50			3	25.00	1	7.14
	Bootstrap	1	3.85	1	16.67			1	8.33		
	Not stated	8	30.77	1	16.67	3	50	4	33.33	4	28.57
**Discount rate**	3%	15	57.69	1	16.67	3	50	4	33.33	11	78.57
	5%	3	11.54	3	50			3	25		
	Not stated	8	30.77	2	33.33	3	50	5	41.67	7	58.33
**Threshold**	3GDP	8	30.77	4	66.67			4	33.33	4	28.57
	1GDP	1	3.85							1	7.14
	140$	2	7.69							2	14.29
	Not stated	15	57.69	2	33.33			8	66.67	7	50
**Guideline**	WHO-CHOICE	6	23.08	4	66.67			4	33.33	2	14.29
	WHO Commission on Macroeconomics and Health	3	11.54							3	21.43
	World Bank’s WorldDevelopment Report1993: Investing in Health	2	7.69							2	14.29
**Fund**	Bill and MelindaGates foundation	4	15.38							4	28.57
	Atlantic Philanthropies	4	15.38	1	16.67			1	8.33	3	21.43
	Other International Funds	5	19.23							5	35.71
	Vietnam Government	1	3.85							1	7.14
**Correspondence Affiliations**	University	17	65.38	3	50	5	83.33	8	66.67	9	64.29
	Government	5	19.23	2	33.33	1	16.67	3	25	2	14.29
	Internationalnon-profit organization	2	7.69							2	14.29
	Others	2	7.69	1	16.67			1	8.33	1	7.14
**Considered the budget impact? (Yes)**		1	3.85	1	16.6667			1	8.33		
**Considered the suitability of ethics, politics, culture and society? (Yes)**		1	3.85			1	16.67	1	8.33		

(Categories of stratification are not mutually exclusive).

The common costing approaches were bottom-up (n = 9), activity-based (n = 7), and top-down (n = 3). The majority of selected studies reported direct cost (n = 24), and only 8 studies considered the indirect cost. Sensitivity analysis was performed in 76.92% studies (n = 20), and included one-way analysis (n = 13), multi-way analysis (n = 2), probabilistic analysis (n = 4), and bootstrap analysis (n = 1). In total, there was only 1 study that considered the budget impact, and only 1 study considered the suitability of ethics, politics, culture and society.

The majority of selected studies (57.69%) used a 3% discount rate, and a plurality (30.77%) used a threshold of 3GDP. Of the studies that stated the use of a particular guideline, the majority followed the WHO-CHOICE guideline (23.08% of total papers), and 57.7% did not state the use of a guideline at all. Approximately half of the studies reported funding from an international source, of which the Bill and Melinda Gates foundation and Atlantic Philanthropies were the most common funders. Only one study was funded by Vietnam Government, and 46.2% of the studies did not state their source of funding. We estimated that nearly three quarters of corresponding authors were affiliated with a university, and only 19.23% were affiliated with the government.

### Cost-effectiveness

**Figure 2:**
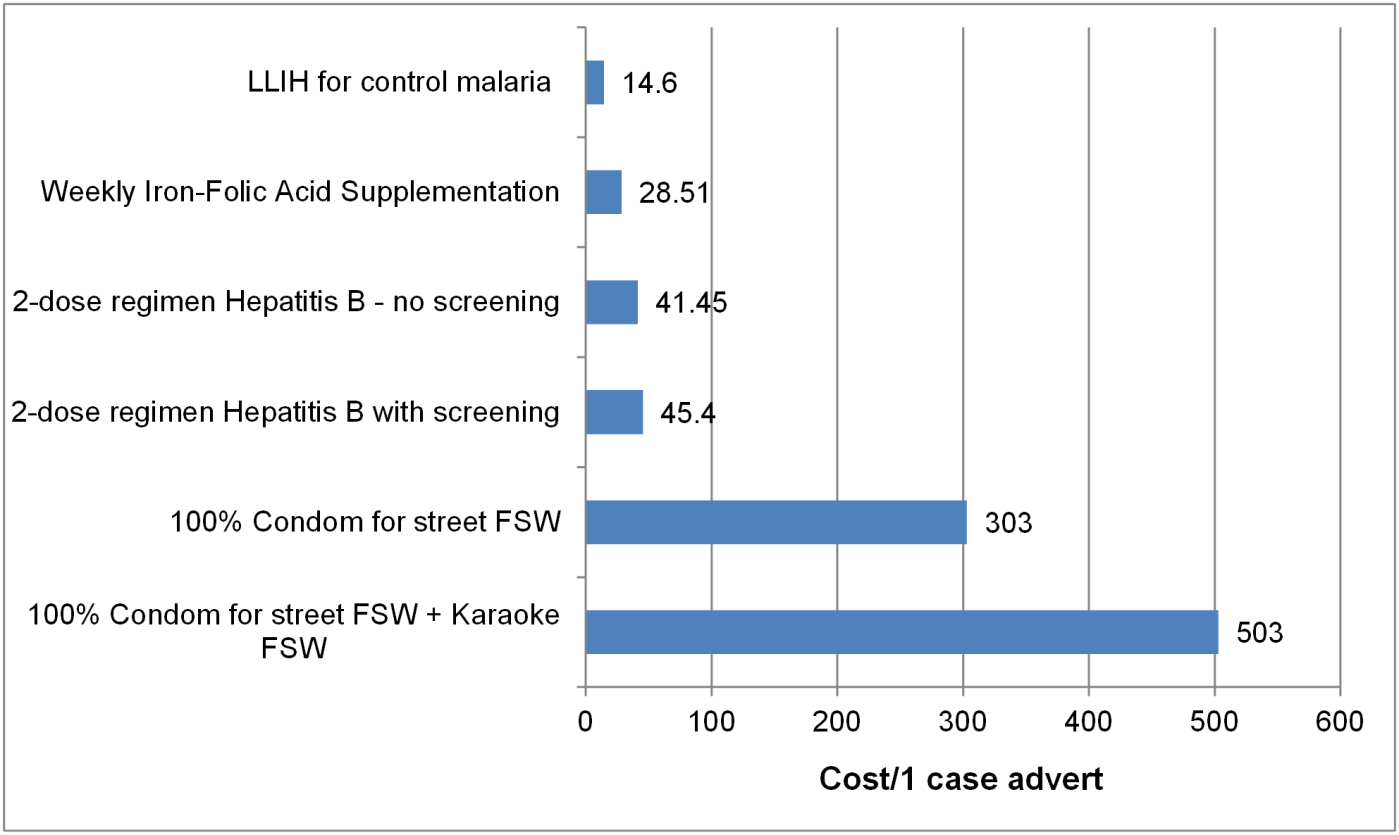
ICERs of studies reported health outcomes by cost per case averted.

**Figure 3:**
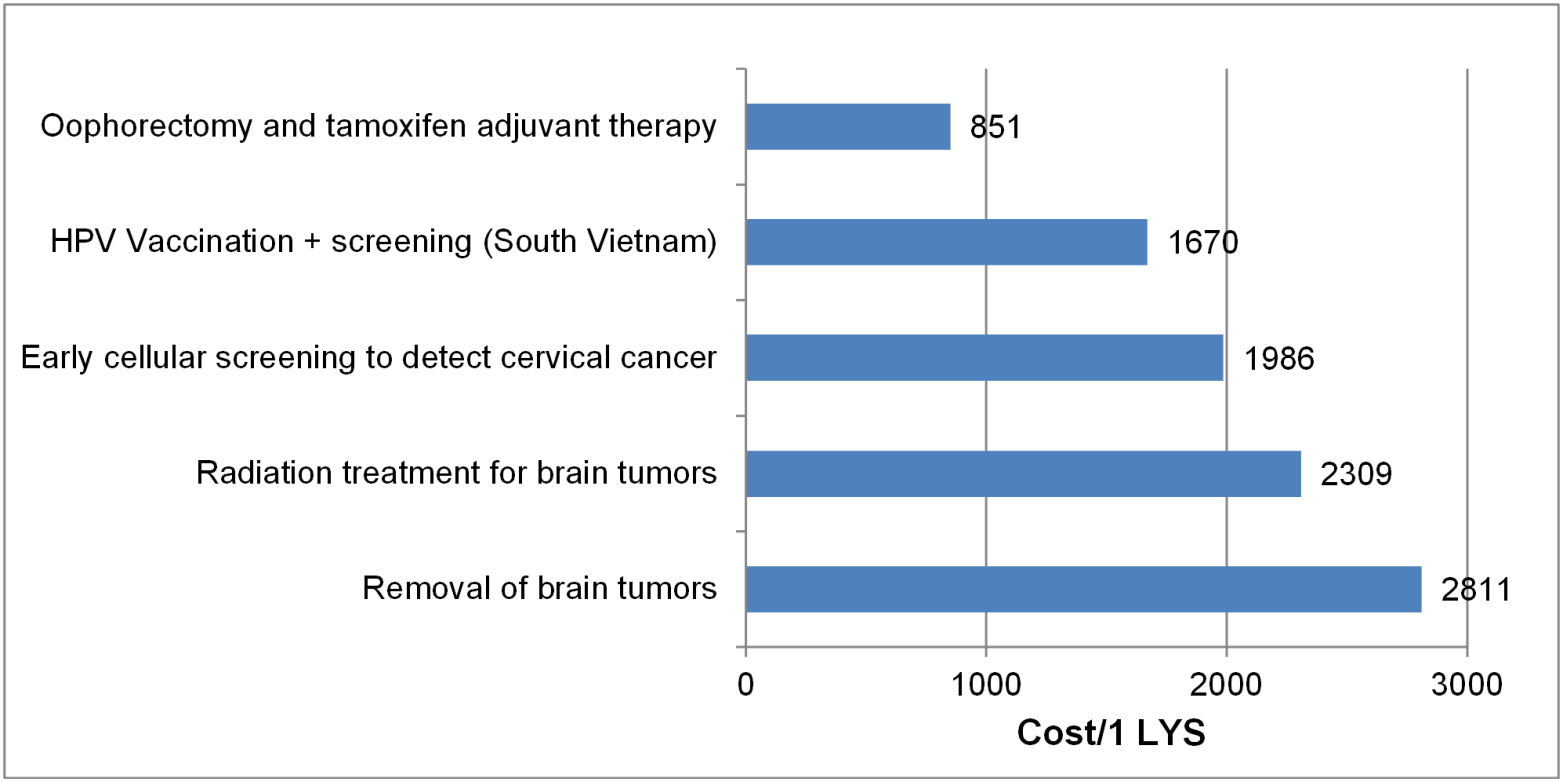
ICERs of studies reported health outcomes by cost per LYS.

We categorized all interventions analyzed in the studies as highly cost-effective, cost-effective, or not cost-effective, depending on how their ICERs compared to GDP per capita in Vietnam. The results of ICERs were shown in **Figure 2** **,** **3** **,** **4** **,** **5** (in these figures, we presented the cost which has been adjusted for inflation). While the results of the studies can obviously not be directly compared, given their differences in calculating and measuring ICERs, we found that most interventions were either highly cost-effective or cost-effective. Among the evaluated studies whose findings were reported in cost per life years saved or cost per case averted (**Figure 2** **and** **figure 3** **),** seven interventions were highly cost-effective, with the ICERs ranging from $US 14.6 to $US 851, which is less than GDP per capita; 4 interventions were cost-effective, with ICERs ranging from $US 1670 to $US 2811, which is between one and three times GDP per capita.

**Figure 4:**
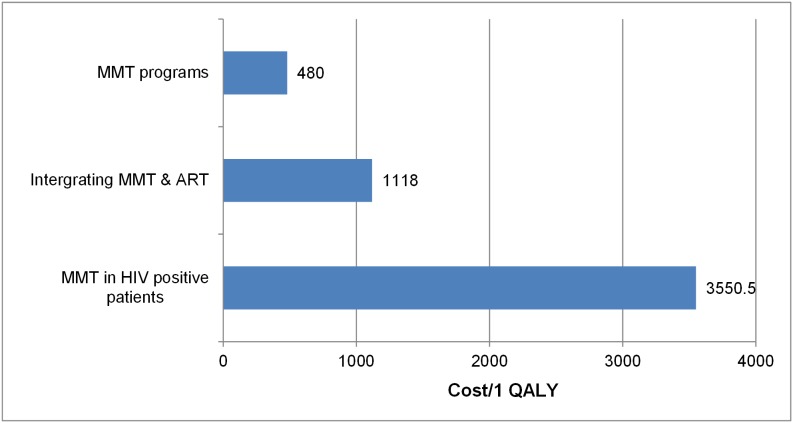
ICERs of studies reporting health outcomes by cost per QALY.

**Figure 5:**
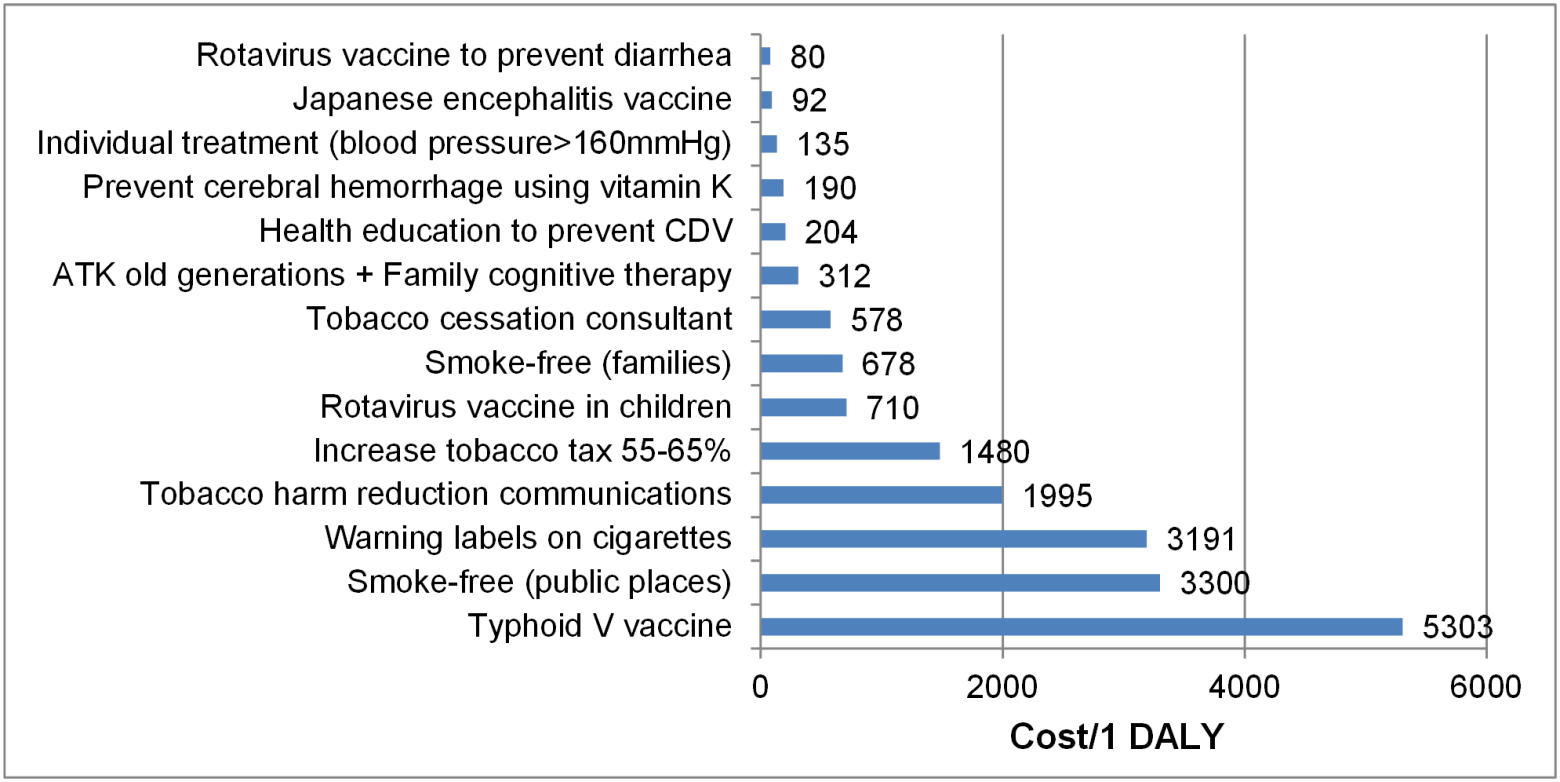
ICERs of studies reporting health outcomes by cost per DALY.

Among studies whose ICERs were measured as DALY or QALY units, we again found that the majority of the interventions analyzed were cost-effective or highly cost-effective (**Figure 4** **and** **figure 5**). Only one intervention-Typhoid V vaccine for children in Hue - was not cost-effective, with an ICER of 5303 $US/DALY, which is more than three times higher than GDP per capita [22]. On the other hand, studies that analyzed methadone maintenance treatment (MMT) programs reported highly cost-effective results, on average 480$/QALY gained. Similarly, of the papers reporting cost per DALY averted, more than half (9) of the studies reported highly cost-effective results, with ICERs ranging from 80 to 1480 $/DALY averted.

### Study Quality

The quality of the evaluated studies was assessed by the Quality of Health Economic Studies instrument. These QHES scores are presented in **Table 5**. In total, 84.6% of the studies evaluated had good or excellent quality scores, and only 15.4% had low quality scores. Among studies conducted by Vietnamese researchers (n = 12), 33.3% of the studies were low quality, with a lowest score was 36. Only 4 studies written by Vietnamese authors had excellent-quality, and all of them were international published. On the other hand, among articles published by international authors, 42.9% were good quality and 57.1% were excellent quality, with a highest score of 100 and no studies with a quality score of less than 75. Finally, when categorized by publication source, 95% of papers published internationally were high or excellent quality compared to 50% of the papers published in Vietnam. These scores were generated based upon the studies’ rating in response to 16 “yes or no” questions (responses shown in **Table 6** **)**. Overall, studies published by Vietnamese researchers had fewer positive responses to the QHES questions than international researchers. Indeed, there were two questions that Vietnamese researchers’ studies had very low score (<50%): “Did the author(s) explicitly discuss direction and magnitude of potential biases?”(Q14) (42%), and “Was there a statement disclosing the source of funding for the study?”(Q16) (8%). A low positive score (50%–60% of the studies) was seen in an additional two questions: “Were the perspectives of the analysis (societal, third-party payer, etc.) and reasons for its selection stated?”(Q2); “Were variable estimates used in the analysis from the best available source (i.e., randomized control trial-best, expert opinion-worst)?”(Q3).

**Table 5:** QHES scores by author nationality and publication source.

		n	QHES score				Classification		
			Mean	SD	Highest	Lowest	<75 (low)	75–90 (high)	>90 (excellent)
**International corresponding authors**	International journals	14	90	8.2	100	77		42.9	57.1
**Vietnamese corresponding authors**	International journals	6	85.7	22	97	42	16.7	16.7	66.7
	Vietnam journals	6	67.3	22.9	87	36	50	50	
	All	12	76.5	23.5	97	36	33.3	33.3	33.3
**Publication source**	International journals	20	88.7	13.3	100	42	5	35	60
	Vietnamese journals	6	67.3	22.9	87	36	50	50	
	**Total**	26	83.8	18	100	36	15.4	38.5	46.2

**Table 6:** Responses to QHES questions.

QHES questions		Positive response to QHES (%)				
		All	Vietnam Corresponding authors			International authors (int journals) (n = 14)
			Int journals (n = 6)	VN journals (n = 6)	ALL (n = 12)	
1	Was the study objective presented in a clear, specific, and measurable manner?	100	100	100	100	100
2	Were the perspective of the analysis (societal, third-party payer, etc.) and reasons for its selection stated?	65	83	17	50	79
3	Were variable estimates used in the analysis from the best available source (i.e., randomized control trial-best, expert opinion-worst)?	65	83	33	58	71
4	If estimates came from a subgroup analysis, were the groups prespecified at the beginning of the study?	96	100	100	100	93
5	Was uncertainty handled by (1) statistical analysis to address random events, (2) sensitivity analysis to cover a range of assumptions?	69	83	50	67	71
6	Was incremental analysis performed between alternatives for resources and costs?	69	100	67	83	57
7	Was the methodology for data abstraction (including the value of health states and other benefits) stated?	96	100	83	92	100
8	Did the analytic horizon allow time for all relevant and important outcomes? Were benefits and costs that went beyond 1 year discounted (3% to 5%) and justification given for the discount rate?	100	100	100	100	100
9	Was the measurement of costs appropriate and the methodology for the estimation of quantities and unit costs clearly described?	88	83	67	75	100
10	Were the primary outcome measure(s) for the economic evaluation clearly stated and did they include the major short-term was justification given for the measures/scales used?	96	83	100	92	100
11	Were the health outcomes measures/scales valid and reliable? If previously tested valid and reliable measures were not available, was justification given for the measures/scales used?	96	83	100	92	100
12	Were the economic model (including structure), study methods and analysis, and the components of the numerator and denominator displayed in a clear, transparent manner?	81	67	67	67	93
13	Were the choice of economic model, main assumptions, and limitations of the study stated and justified?	88	83	67	75	100
14	Did the author(s) explicitly discuss direction and magnitude of potential biases?	65	83	0	42	86
15	Were the conclusions/recommendations of the study justified and based on the study results?	100	100	100	100	100
16	Was there a statement disclosing the source of funding for the study?	54	17	0	8	93

Among articles from international researchers, the majority of studies were scored favorably for the QHES criteria questions; more than 80% of these studies were evaluated as “Yes” for 13 of the 16 questions. The lowest scored question, with only 57% of the studies scored positively, was Q6, “Was incremental analysis performed between alternatives for resources and costs?” Questions that were scored only moderately, with 70–75% of the studies scoring positively, were Q3, and Q5: “Were variable estimates used in the analysis from the best available source (i.e., randomized control trial-best, expert opinion-worst)?”; and “Was uncertainty handled by (1) statistical analysis to address random events, (2) sensitivity analysis to cover a range of assumptions?

## Discussion

Health economic evaluation has been developed and increasingly implemented over the past few decades in order to support resource-allocation decisions. However, despite the limited funding and growing healthcare spending needs in Vietnam, there has yet to be established a formal health economic evaluation process in this country, and very little research exists in this field. We identified only 26 health economic evaluations to include in this review, 20 published internationally and 6 published in Vietnam. Given the small number of papers, the topics they covered are obviously quite limited. A slight majority focused on infectious diseases, with HIV being the most common topic among them. It is interesting to compare this to the disease burden in Vietnam, where 56% of life years lost is due to non-communicable diseases and only 29% are due to communicable diseases (WHO Vietnam health profile). Indeed, 40% of mortality in Vietnam is due to cardiovascular disease, yet only one study included in this review examined this topic.

The limited number and scope of the topics indicates that demands for health economic research to inform policy development remains limited. If the ultimate goal of health-economic evaluation is to support evidence-based policy decisions, then it is essential that the research being produced is of interest to the decision makers. This suggests the need for more institutional links between the producers (researchers) and end-users (policy-makers) of the research. Many countries, such as Thailand, Malaysia, South Korea, and Taiwan, have had success with this model, by requiring cost-effectiveness data in order for pharmaceutical products to be authorized for the local market, or to be eligible for reimbursement [35], [36]. Even China, which does not have socialized health care like the previous examples, was actually the first country to implement such a formal process, as a means to efficiently utilize healthcare resources in a fee-for-service system that incentivizes doctors to overprescribe medicine and technology [35]. The important commonality of all these examples is that the demand for research comes from the policy-makers themselves, ensuring that the evidence produced is highly relevant to policy makers. While this study did not include any unpublished material, such as government documents, a formal process for cost-effectiveness analysis does not currently exist in Vietnam [35].

When we assessed the study quality, we found that the studies’ quality was constrained by certain technical limitations, including lack of sensitivity analysis, using low-quality data sources, and failing to report things like funding sources, potential sources of bias, and the perspective of the cost analysis. These limitations are problematic in that they reduce the credibility of the research. This finding indicated that health economic research has some major technical limitations is reflected in other developing countries, including India, South Africa, and Nigeria [7]–[9]. However, unlike these studies, which all found that papers authored by foreign authors had higher quality than those produced by local authors, we found that although the Vietnamese authored papers were generally of lower quality than the foreign authored papers, there was a greater difference in quality based on publishing location than on nationality of author. Papers published internationally and written by Vietnamese authors had an average quality score of 85.7, compared to a score of 90 for internationally published papers by foreign authors.

On the one hand, this difference in quality based on publication location should be expected. Naturally, authors who produce papers of higher quality would be interested in publishing their papers in international journals. However, this finding highlights a couple of areas for concern. First, only 50% of the Vietnamese authors included in this study published in international journals, which suggests that barriers including language and research capacity may be inhibiting many Vietnamese authors from publishing in international journals. In order to ensure that Vietnamese researchers have the capacity to produce high quality health economic research, such research techniques should be incorporated into medical and public health education in Vietnam.

Second, the fact that the bulk of high quality health economic research about Vietnam has been published internationally suggests that the target audience of this research is international, not domestic. Combined with the fact that of the 14 papers that reported funding information, 13 were funded by international sources, and only 1 by the Vietnamese government, this information calls into question whose interests are being addressed by the current research. In order for health economic research to have an impact on local policy decisions, it must be locally relevant, and if research about a country is too heavily dictated by foreign interests, the research produced may be less likely to align with the local political context and culture, and thus less likely to be relevant to policy makers [2]. This again suggests that there needs to be more institutional links between policy makers and researchers, so that in the future, the research produced can be more targeted to a local audience.

In addition, the quality of the locally produced research should be improved, not only through improved research capacity, but also through enforcing the use research guidelines. Indeed, 100% of the papers published in Vietnam did not report using any HEE guidelines for their study. Many of the technical limitations that currently exist in many of the studies published in Vietnamese journals may be resolved if the studies had to comply to cost-effectiveness analysis guidelines. An example of successful implementation of national guidelines for health economic research comes from South Korea, which allows industry to produce cost-effectiveness research, but manages the quality and credibility of this research by issuing guidelines [36].

While this study used the QHES instrument, which has been found to be an accurate assessment of health economic study quality [4], it should be noted that this review paper may have some limitations. Since many Vietnamese databases are incomplete, it is possible that some relevant Vietnamese studies were not included in the review. Additionally, some research may have been excluded due to publication bias itself, since studies with positive results are more likely to be published that those with negative results. Therefore, this review excluded from analysis many studies with negative results, or with too low of quality to be published. The scope of this review was limited to published, academic papers only, so much of the body of writing about healthcare in Vietnam – including press conferences, pharmaceutical company announcements, and government reports - was not selected for review. Finally, the lack of clarity in many of the papers’ descriptions of methodology and results may have impaired our process of classification and analysis.

## Conclusions

In order to address the country’s pressing healthcare needs, Vietnam must mobilize its limited resources as efficiently as possible. Health economic evidence can play a key role in such decisions about resource allocation, however we identified several technical and institutional limitations with the current research. In order to improve the relevancy of research to policy-makers, Vietnam should implement a formal process of health economic research that involves communication between researchers, policy-makers, and health practitioners. Additionally, the quality of health economic research should be improved both through education and the implementation of research guidelines for national research.

## Supporting Information

Table S1 Summary Profile of selected studies.
(DOCX)

Checklist S1 PRISMA Checklist.
(DOC)
