# Supplementary material for: A Systematic Review of Scope and Quality of Health Economic Evaluation Studies in Vietnam
Source: PLoS One. 2014 Aug 14;9(8):e103825. doi: 10.1371/journal.pone.0103825 (PMC4133226; doi:10.1371/journal.pone.0103825)
Supplement: Table S1 — Summary Profile of selected studies. (DOCX) [file pone.0103825.s001.docx]

**Table S1. Summary Profile of selected studies.**

| **Study (Year)** | **Type** | **Design** | **Type of disease** | **Perspective** | **Outcomes** | **Objectives** | **Results** | **Discount rate/ Threshold** | **Guidelines Followed** | **Funding** | **Correspondences/** **Affiliations** |
| --- | --- | --- | --- | --- | --- | --- | --- | --- | --- | --- | --- |
| Chantal M. Morel 2013  [1] | CEA | RCT | Malaria | Societal, Health system | Case averted | Assess the cost-effectiveness of using long-lasting insecticide-treated hammocks in Ninh Thuan | Average savings per episode averted were estimated to be $ 14.60 USD for the health system and 14.37 USD for households (including both direct and indirect cost savings) | DR: 3% |  | Belgium Corporation | International researcher/ LSE Health, London School of Economics & Political Science |
| Gerard J. Casey 2011  [2] | CEA |  | Anaemia | Health system | LYS | Estimate the cost and cost-effectiveness of a project administering deworming and weekly iron-folic acid supplementation to control anaemia in women of reproductive age in Yen Bai province, Vietnam | The cost per life year gained, was equal to 46.4 million VND (US$2811) for SR compared to 38.1 million VND (US$2309) for SRS | DR: 0% |  | The Atlantic Philanthropies Incorporated | International researcher/ Department of Medicine (RMH/WH), The University of Melbourne |
| Vivien D Tsu 2009  [3] | CEA |  | Postpartum hemorrhage | Health system | Case averted | Present data on the costs and cost-effectiveness of introducing the routine use of active management of third-stage labour for the health system in Vietnam. | $15.70 - $21.68 per case averted | DR: 3% |  | Bill & Melinda Gates Foundation | International researcher/ PATH, Seattle, WA, USA |
| Bach Tran 2013  [4] | CEA | Modeling | HIV/AIDS | Health system | QALY | Evaluate the cost-effectiveness of MMT for HIV prevention and treatment among opioid dependents, and to analyze the budget impact of scaling up the MMT program from 2011 to 2015 in Vietnam | The cost-effectiveness ratio of MMT and non-MMT strategies was US$480 and US$204 per 1 quality-adjusted life year (QALY) | DR: 5%; threshold: 3GPD | WHO-CHOICE |  | Vietnamese researcher/Department of Public Health Sciences, School of Public Health , University of Alberta |
| Jane J. Kim 2008  [5] | CEA | Modeling | HPV | Societal | LYS | Assess the cost-effectiveness of cervical cancer prevention strategies and the tradeoffs between a national and regional-based policy in Vietnam | In North Vietnam, screening + HPV Vaccine (5 year): $ 7,250 / LYS; South Vietnam: Screening + HPV Vaccine (5 year): $ 1,190 / YLS | DR: 3%; threshold: 1xGDP; 0.5xGDP | WHO Commission on Macroeconomics and Health | Bill and Melinda Gates Foundation | International researcher/ Department of Health Policy and Management, Program in Health Decision Science, Harvard School of Public Health |
| Duong Anh Vuong 2012  [6] | CEA | Retrospective database analysis | Brain Metastasis | Service users | LYS | Evaluate the cost-effectiveness of the treatment of brain metastasis with surgical resection (SR) and stereotactic radiosurgery (SRS) in the lower-middle-income country of Vietnam from the perspective of patients and families | The cost per life year gained, was equal to 46.4 million VND (US$2811) for SR compared to 38.1 million VND (US$2309) for SRS |  |  |  | Vietnamese researcher/Department of Medical Service Administration, Ministry of Health of Vietnam |
| Richard R. Love 2002 [7] | CEA | Controlled clinical trial | Breast Cancer |  | LYS |  | $350 per life-year saved. | DR: 3% |  | US: National  Institutes of Health, University of  Wisconsin Clinical Cancer Center, International Breast  Cancer Research Foundation | International researcher/ University of Wisconsin Comprehensive Cancer Center |
| Eric J. Suba 2001 [8] | CEA | Modeling | Cervical cancer | Societal | LYS | Assess the cost-effectiveness of Papanicolaou Cytology Screening Services in Vietnam | 628-785$/Discounted Life Year gained | DR: 3% |  | Department of Health  Services, Ho Chi Minh City, and the Ministry of  Health | International researcher/ Kaiser Permanente Medical Center, Redwood |
| Bach Tran 2012 [9] | CEA | Modeling | HIV/AIDS | Health system | QALY | Evaluate the cost-effectiveness of integrating MMT with ART for HIV-positive DUs in Vietnam | The cost-effectiveness ratios of ART, ART-MMT, and DAART-MMT strategies were 1,358, 1,328, and 1,118 (USD/ QALY) | DR: 5%; threshold: 3GPD | WHO-CHOICE |  | Vietnamese researcher/Department of Public Health Sciences, School of Public Health , University of Alberta |
| Hideki Higashi 2012 [10] | CEA | Modeling | Smoking | Health system | DALY | Examine the cost-effectiveness of personal smoking cessation support in Vietnam | Physician advice: international dollars 543, per DALY averted; pharmacological therapies are not cost-effective | DR: 3%; threshold: 3GDP | WHO Commission on Macroeconomics and Health | Atlantic Philanthropies | International researcher/ The University of Queensland, School of Population Health |
| Duc Anh Ha 2010 [11] | CEA | Modeling | CDV | Societal | DALY | Assess the cost-effectiveness of a set of personal and non-personal prevention strategies to reduce cardiovascular disease in Vietnam | A health education program to reduce salt intake (VND 1 945 002 or US$118 per DALY averted) and individual treatment of systolic blood pressure above 160 mmHg (VND 1 281 596 or US$78 per DALY averted) | DR: 3%; threshold: 3GDP | WHO-CHOICE | Vietnam  Education Foundation, Jiaikai Foundation, Atlantic Philanthropies | Vietnamese researcher/Vietnam Ministry of Health |
| Sun-Young Kim 2009 [12] | CEA | Modeling | Diarrhea | Societal, Health system | DALY | Evaluate the cost-effectiveness of a rotavirus vaccination program in Vietnam | The incremental cost per DALY averted from vaccination compared to no vaccination was $540 from the societal perspective and $550 from the health care system perspective | DR: 3%; threshold: 3GDP | WHO-CHOICE | Bill and Melinda Gates Foundation | International researcher/ Department of Health Policy and Management, Harvard School of Public Health |
| Joseph Cook 2008 [13] | CEA | Modeling | Typhoid V | Societal, Third-Party Payer | DALY | Evaluate the cost-effectiveness of Typhoid V vaccination against typhoid in sites in four Asian cities: Kolkata (India), Karachi (Pakistan), North Jakarta (Indonesia), and Hue (Vietnam) | A program targeting school-aged children in Hue, Vietnam would prevent 21 cases, avert 6 DALYs, ICERs: US$3779 per DALY averted | DR: 3%; threshold: 3GNI | WHO-CHOICE | Bill and Melinda Gates Foundation | International researcher/Evans School of Public Affairs, University of Washington |
| Thea K. Fischer 2005  [14] | CEA | Modeling | Diarrhea | Societal, Health system | DALY | Assess vaccine cost-effectiveness in Vietnam | At a price of $5 - $20/vaccine course, the cost per DALY averted is $40 - $192 | DR: 3%; threshold: $140/DALY | World Bank’s World Development Report 1993: Investing in Health | Program for Appropriate Technology in Health; Global Alliance for Vaccines and Immunization; Vaccine Fund; US CDC | International researcher/Division of Viral and Rickettsial Diseases and Epidemiology Intelligence Service, Centers for Disease Control and Prevention |
| N Danielsson 2004 [15] | CEA | Retrospective database analysis | Hemorrhage |  | DALY | Estimate the burden of intracranial hemorrhage caused by late onset vitamin K deficiency bleeding in Hanoi, Vietnam | Routine vitamin K prophylaxis would significantly reduce infant morbidity and mortality in Vietnam and cost an estimated US$87 (£48, J 72) per DALY | DR: 3%; threshold: 140$/DALY | World Bank’s World Development Report 1993: Investing in Health | Royal Children’s Hospital International, WHO | International researcher/Department of Neonatology, Royal Children’s Hospital, Australia |
| Charles B. Holmes 2010  [16] | CEA | Retrospective database analysis | HIV/AIDS |  | Cost savings | Examine trends in the volume, costs, and types of ARVs purchased with PEPFAR funds since the introduction of the FDA tentative approval process, and to estimate the cost savings achieved through the use of generic drugs from 2005 through 2008 | Estimated yearly savings generated through generic ARV use were $8 108 444 in 2005, $24 940 014 in 2006, $75 645 816 in 2007, and $214 648 982 in 2008, a total estimated savings of $323 343 256. |  | PEPFAR |  | International researcher/Office of the US Global AIDS Coordinator, US Department of State |
| Bach Tran 2012 [17] | CEA | Modeling | HIV/AIDS | Health system | QALY | Assess the incremental cost-effectiveness of a multi-site MMT program for HIV-positive drug users in Vietnam from the health service provider perspective | The overall ICER per 1 QALY gained was $ 3,550.5 | DR: 5%; threshold: 3GPD | WHO-CHOICE |  | Vietnamese researcher/Department of Public Health Sciences, School of Public Health , University of Alberta |
| Hideki Higashi 2011 [18] | CEA | Modeling | Smoking | Health system | DALY | Analyze the cost effectiveness of four population-level tobacco control interventions in Vietnam | Tax increase from 55% to 65%: 1390; Tax increase from 55% to 75%: 2788; Tax increase from 55% to 85%: 4050; Graphic pack warning label: 2996; Mass media campaign: 1873; Smoking ban (public): 3099; Smoking ban (work): 637/ DALY averted | DR: 3%; threshold: 3GDP | WHO Commission on Macroeconomics and Health | Atlantic Philanthropies | International researcher/ The University of Queensland, School of Population Health |
| Nguyen Thi My Huong 2001 [19] | CBA |  | Reproduction |  | Cost savings | Analyze the cost savings of urine pregnancy tests prior to menstrual regulation in Vietnam | The total savings per annum resulting from routine urine pregnancy testing is $2 023 770 |  |  |  | Vietnamese researcher |
| A. Tyrell [26] | CEA |  | Anaemia | Health system | Cost/effectiveness test | To compare the cost and effectiveness of Copper Sulphate (CS) and HemoCue (HC) methods for screening blood donors for anaemia | Even at a very high probability of an ineffective CS test, the cost of the microcuvette will need to be significantly lower than its current cost in order for the HC test to become less costly than CS per effective test achieved |  |  |  | International researcher/ Liverpool School of Tropical Medicine |
| Nguyen Thu Yen 2006 [20] | CEA |  | Japanese encephalitis |  | Case averted/ DALY averted | Evaluate the cost-effectiveness of a national vaccine program against Japanese encephalitis | $ 465/case advert, $ 49/DALY advert | DR: 3% |  |  | Vietnamese researcher/Vietnam National Institute Of Hygiene And Epidemiology |
| Nguyen Thu Ha 2012 [21] | CEA |  | Schizophrenia |  | DALY | Analyze the cost - effectiveness of interventions for schizophrenia patients in Vietnam | Older ATK generation + family cognitive therapy : 6545.718 VNĐ /DALY, older ATK generation (alone): 6470.804 VNĐ/DALY | DR: 3% |  |  | Vietnamese researcher/Hanoi School of Public Health |
| Le The Thu 2003 [22] | CEA |  | Hepatitis B |  | Case advert | Analyze the cost-effectiveness of two method: screening and not screening hepatitis B | 2-dose regimen with no screening: 279,500 VND /case averted and 2-dose regimen screening: 306,060 VND /case averted |  |  |  | Vietnamese researcher/Ho Chi Minh Medicine And Pharmacy University |
| Vu Duy Kien 2012 [23] | CEA |  | HIV/AIDS |  | Case averted | Evaluate the cost-effectiveness of condom distribution for female sex workers in An Giang province | Female condom intervention for Street FSWs: $ 303/ HIV case averted, combined Street FSWs and Karaoke FSWs: $ 503 / HIV case averted |  |  |  | Vietnamese researcher/Hanoi Medical University |
| Cao Ngoc Nga 2013 [24] | CEA |  | Hepatitis B |  | Case avert | Assess the cost-effectiveness of two methods: screening and not screening hepatitis B | 4866391 VNĐ/case averted (29-39 years old); 6988166 VNĐ/case averted (40 years old) |  |  |  | Vietnamese researcher/Ho Chi Minh Medicine And Pharmacy University |
| Nguyen Dinh Son 2009 [25] | CEA |  | EPI |  | Cost savings | Estimate the cost savings of Expanded Program on Immunization in Vietnam | Cost savings at Phu Loc: $ 31,338.960, Nam Dong: $ 703.440 | DR: 3% |  |  | Vietnamese researcher/Hue School of Medicine |

**References**

1. Morel CM, Thang ND, Erhart A, Xa NX, Peeters Grietens K, et al. (2013) Cost-effectiveness of long lasting insecticide-treated hammocks in preventing malaria in South-central Vietnam. PLoS One8: e58205.
2. Casey GJ, Sartori D, Horton SE, Phuc TQ, Phu LB, et al. (2011) Weekly iron-folic acid supple mentation with regular deworming is cost-effective in preventing anaemia in women of reproductive age in Vietnam. PLoS One 6: e23723.
3. Tsu VD, Levin C, Tran MP, Hoang MV, Luu HT (2009) Cost-effectiveness analysis of active management of third-stage labour in Vietnam. Health Policy Plan 24: 438-444.
4. Tran BX, Ohinmaa A, Duong AT, Nguyen LT, Vu PX, et al. (2012) The cost-effectiveness and budget impact of Vietnam's methadone maintenance treatment programme in HIV prevention and treatment among injection drug users. Global Public Health 7: 1080-1094.
5. Kim JJ, Kobus KE, Diaz M, O'Shea M, Van Minh H, et al. (2008) Exploring the cost-effectiveness of HPV vaccination in Vietnam: insights for evidence-based cervical cancer prevention policy. Vaccine 26: 4015-4024.
6. Vuong DA, Rades D, Le AN, Busse R (2012) The cost-effectiveness of stereotactic radiosurgery versus surgical resection in the treatment of brain metastasis in Vietnam from the perspective of patients and families. World Neurosurg 77: 321-328.
7. Love RR, Duc NB, Allred DC, Binh NC, Dinh NV, et al. (2002) Oophorectomy and tamoxifen adjuvant therapy in premenopausal Vietnamese and Chinese women with operable breast cancer. J Clin Oncol 20: 2559-2566.
8. Suba EJ, Nguyen CH, Nguyen BD, Raab SS (2001) De novo establishment and cost-effectiveness of Papanicolaou cytology screening services in the Socialist Republic of Vietnam. Cancer 91: 928-939.
9. Tran BX, Ohinmaa A, Duong AT, Nguyen LT, Vu PX, et al. (2012) Cost-effectiveness of integrating methadone maintenance and antiretroviral treatment for HIV-positive drug users in Vietnam's injection-driven HIV epidemics. Drug Alcohol Depend 125: 260-266.
10. Higashi H, Barendregt JJ (2012) Cost-effectiveness of tobacco control policies in Vietnam: the case of personal smoking cessation support. Addiction 107: 658-670.
11. Ha DA, Chisholm D (2011) Cost-effectiveness analysis of interventions to prevent cardiovascular disease in Vietnam. Health Policy Plan 26: 210-222.
12. Kim SY, Goldie SJ, Salomon JA (2009) Cost-effectiveness of Rotavirus vaccination in Vietnam. BMC Public Health 9: 29.
13. Cook J, Jeuland M, Whittington D, Poulos C, Clemens J, et al. (2008) The cost-effectiveness of typhoid Vi vaccination programs: calculations for four urban sites in four Asian countries. Vaccine 26: 6305-6316.
14. Fischer TK, Anh DD, Antil L, Cat ND, Kilgore PE, et al. (2005) Health care costs of diarrheal disease and estimates of the cost-effectiveness of rotavirus vaccination in Vietnam. J Infect Dis 192: 1720-1726.
15. Danielsson N, Hoa DP, Thang NV, Vos T, Loughnan PM (2004) Intracranial haemorrhage due to late onset vitamin K deficiency bleeding in Hanoi province, Vietnam. Arch Dis Child Fetal Neonatal Ed 89: F546-550.
16. Holmes CB, Coggin W, Jamieson D, Mihm H, Granich R, et al. (2010) Use of generic antiretroviral agents and cost savings in PEPFAR treatment programs. JAMA 304: 313-320.
17. Tran BX, Ohinmaa A, Duong AT, Do NT, Nguyen LT, et al. (2012) Cost-effectiveness of methadone maintenance treatment for HIV-positive drug users in Vietnam. AIDS Care 24: 283-290.
18. Higashi H, Truong KD, Barendregt JJ, Nguyen PK, Vuong ML, et al. (2011) Cost effectiveness of tobacco control policies in Vietnam: the case of population-level interventions. Appl Health Econ Health Policy 9: 183-196.
19. Huong NT, Chongsuvivatwong V, Geater A, Prateepchaikul L (2001) Cost-benefit analysis of urine pregnancy tests prior to menstrual regulation in Vietnam. Am J Public Health 91: 825-826.
20. Yến NT, Kien VD, Anh NPL (2006) Chi phi - hiệu quả của tiem vacxin để kiểm soat bệnh viem nao Nhật Bản tại Việt Nam. Y học dự phong XVI-1: 32-36.
21. Ha NT, Anh NQ, Linh BN, Hương NT (2012) Phan tich chi phi - hiệu quả của cac can thiệp điều trị bệnh tam thần phan liệt tại Việt Nam. Y học dự phong XXII - 2: 54-59.
22. Thự LT, Nga CN (2003) Phan tich chi phi, hiệu quả trong chủng ngừa viem gan sieu vi B ở nhom tuổi 10-14 tuổi khi sử dụng phac đồ 2 liều vaccin. Y học thực hanh 11: 38-41.
23. Kien VD, Minh HV (2012) Chi phi hiệu quả của chương trinh 100% bao cao su nhằm phong chống lay truyền HIV cho nhom gai mai dam tại tỉnh An Giang. Tạp chi nghien cứu khoa học 79: 127-131.
24. Nga CN, Thự LT (2003) Phan tich chi phi, hiệu quả hai phương phap sang lọc va khong sang lọc trong chủng ngừa viem gan sieu vi B Y học thực hanh 8: 62-66.
25. Sơn NĐ (2008) Nghien cứu chi phi-hiệu quả chương trinh tiêm chủng mở rộng tại huyện đồng bằngPhu Lộc va huyện miền nui Nam Đong tại tỉnh Thừa Thien Huế năm 2008. Y học dự phong XIX - 2:116 - 122.
26. Tyrrell, A., et al., Cost and effectiveness comparison of two methods for screening potential blood donors for anaemia in Vietnam. Transfus Med, 2011. 21(3): p. 158-65.
